# A Case Report of an Enlarged Suboccipital Nerve with Cutaneous Branch

**DOI:** 10.7759/cureus.2933

**Published:** 2018-07-06

**Authors:** Sasha Lake, Joe Iwanaga, Rod J Oskouian, Marios Loukas, R. Shane Tubbs

**Affiliations:** 1 Anatomical Studies, St. George's, St. George, GRD; 2 Medical Education and Simulation, Seattle Science Foundation, Seattle, USA; 3 Neurosurgery, Swedish Neuroscience Institute, Seattle, USA; 4 Anatomical Sciences, St. George's University, St. George's, GRD; 5 Neurosurgery, Seattle Science Foundation, Seattle, USA

**Keywords:** suboccipital nerve, c1 nerve, occiput cutaneous innervation, sensory suboccipital nerve

## Abstract

Variations of the suboccipital nerve are infrequently reported. This nerve derived from the C1 spinal nerve is usually a small branch that primarily innervates the short suboccipital muscles. During the routine dissection of the occipital region in an adult cadaver, a vastly enlarged left-sided suboccipital nerve was identified. The nerve innervated the short suboccipital muscles and overlying semispinalis capitis in normal fashion. However, it continued cranially to end in the overlying skin of the occiput. Although not normally thought to have a cutaneous branch, recalcitrant occipital neuralgia might be due to such a variant branch. Future studies are necessary to further elucidate this proposed pathomechanism.

## Introduction

The suboccipital nerve is the dorsal ramus of C1. This nerve is found between the skull and atlas and within the suboccipital triangle. Here, it is positioned between the posterior arch of the atlas and vertebral artery bordering the nerve inferiorly and superiorly, respectively [1]. The suboccipital nerve innervates the rectus capitis posterior major and minor, obliquus capitis superior, obliquus capitis inferior, and semispinalis capitis.

A spinal nerve’s dorsal root and dorsal ganglion carry sensory fibers to the spinal cord. Interestingly, a dorsal root ganglion for C1 has been reported to be found in less than 10% of dissections [2]. However, on a microscopic level, collections of sensory neurons have been identified along the accessory nerve in embryos and adult human cadavers with some positing that these represent cells of the dorsal root ganglion of C1. Neuronal collections have also been visualized grossly along the accessory nerve [2]. Moreover, from a gross perspective the dorsal root ganglion may not be identified grossly even when the dorsal root is present [3], but ectopic neurons can be microscopically seen [4]. The function of the suboccipital nerve is understood to be primarily motor, and not surprisingly, the C1 ventral root is larger compared to its dorsal root—a characteristic that is unique to this nerve compared to other spinal nerves [3]. Taken together, the sensory innervation of the suboccipital nerve is not well understood [3].

## Case presentation

**Figure 1:**
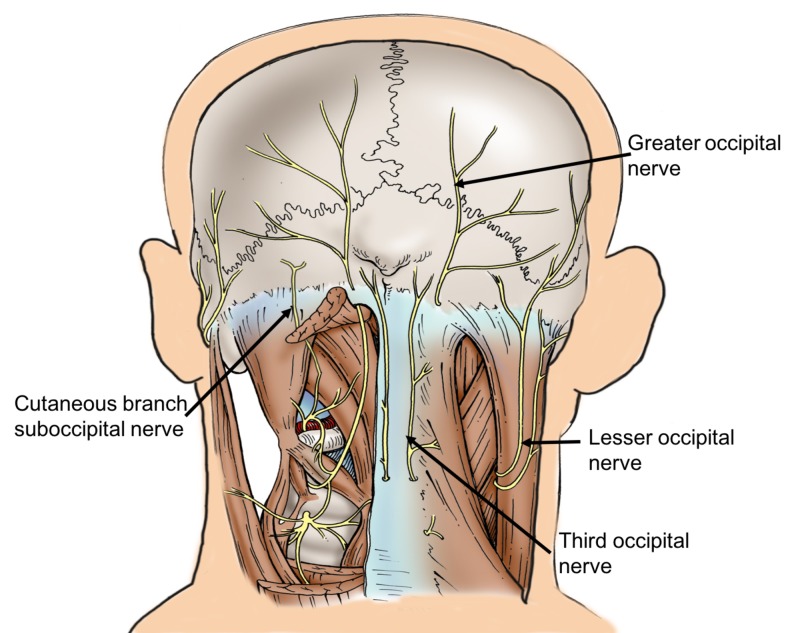
Schematic drawing of the specimen reported herein. Note the variant cutaneous branch of the suboccipital nerve on the left side.

**Figure 2:**
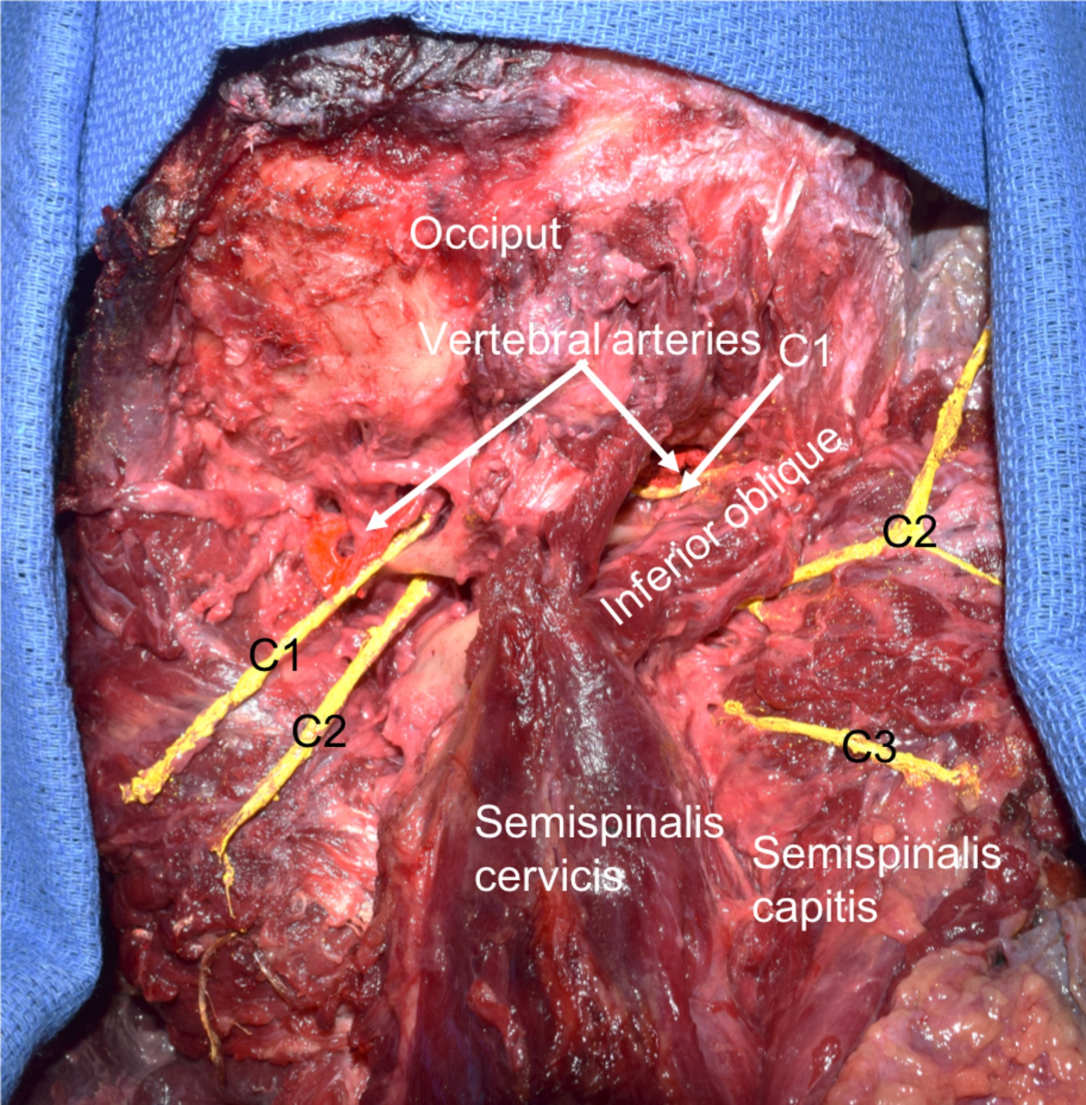
Cadaveric specimen with dissection of the suboccipital region. Note the large and long dorsal ramus of C1 on the left and compare this to the size of the normal dorsal ramus of C1 on the right side. For reference, the semispinalis capitis muscle is reflected laterally on both sides. Also observe the relationship of the suboccipital nerves and the vertebral arteries as they course over the posterior arch of the atlas.

During the routine dissection of the occipital region in an adult cadaver, a vastly (approximately four times the size of the contralateral suboccipital nerve) enlarged left-sided suboccipital nerve was identified (Figures 1, 2). The suboccipital nerve on the left side coursed just medial to the vertebral artery at the posterior arch of the atlas. The nerve innervated the short suboccipital muscles and overlying semispinalis capitis in normal fashion. However, it continued cranially to end in the overlying skin of the occiput by piercing the overlying trapezius muscle. The patch of skin was located below the lambdoid suture at the level of the inion (Figure 1). No articular branches were identified arising from the suboccipital nerve. The right-sided suboccipital nerve was found to have a typical size and length and did not have a cutaneous branch. No other regional anatomical variations or pathology were identified.

## Discussion

The accessory nerve and C1 can be found to have anastomoses. Type I is described as a formation of C1 spinal nerve from ventral roots, but lacking dorsal roots and having an anastomosis with the accessory nerve. Typical formation of spinal nerves, where the nerve is formed by both dorsal and ventral roots, represents a type II neural interconnection without an anastomosis between C1 and the accessory nerve. Type III describes a nerve (Mackenzie’s nerve) connecting the C1 nerve to the accessory nerve. Under type III classification, the suboccipital nerve also has typical spinal nerve formation. Lastly, type IV represents a C1 nerve that lacks dorsal rootlets with anastomotic connection with the accessory nerve and ventral roots of C1 nerve, via Mackenzie’s nerve.

On a microscopic level, collections of sensory neurons of the C1 nerve have been consistently localized coursing along the accessory nerve in embryos and adult human cadavers. These neuronal collections can also be appreciated grossly along the accessory nerve. The ganglia have been theorized to be involved in muscle spindle fiber proprioception but not cutaneous innervation [4].

Although not normally thought to have a cutaneous branch, recalcitrant occipital neuralgia might be due to such a variant branch of the suboccipital nerve as reported herein [5,6]. Additionally, studies aimed at tracing these cutaneous pathways when a C1 dorsal ganglion or root does not exist will add to our knowledge of this spinal nerves’ frequent connections to the accessory nerve [7,8]. Additionally, with improved imaging of the neck muscles, newer technologies might better illustrate the nerves of this area [9]. Although Bergman et al. [10] have mentioned that this nerve “occasionally supplies a cutaneous branch to the back of the head,” further anatomical studies are needed to quantitate this variation and help precisely localize the skin innervated by the nerve.

## Conclusions

Future studies are necessary to further elucidate the anatomy of a cutaneous branch of the suboccipital nerve including its prevalence. Until then, clinicians who treat patients with occipital pain might consider the possibility of a cutaneous contribution to this area from the suboccipital nerve.
